# Portable Devices for Measurement of Vitamin A Concentrations in Edible Oil: Field Readiness of Available Options

**DOI:** 10.1021/acsomega.1c07181

**Published:** 2022-05-17

**Authors:** Samantha L. Huey, Jesse T. Krisher, David Morgan, Penjani Mkambula, Balaji Srinivasan, Bryan M. Gannon, Mduduzi N. N. Mbuya, Saurabh Mehta

**Affiliations:** † Division of Nutritional Sciences, Cornell University, Ithaca, New York 14853, United States; ‡ Department of Large Scale Food Fortification, The Global Alliance for Improved Nutrition, 1202 Geneva, Switzerland; § The Global Alliance for Improved Nutrition, Washington, D.C. 20036, United States; ∥ Institute for Nutritional Sciences, Global Health, and Technology (INSiGHT), Cornell University, Ithaca, New York 14853, United States

## Abstract

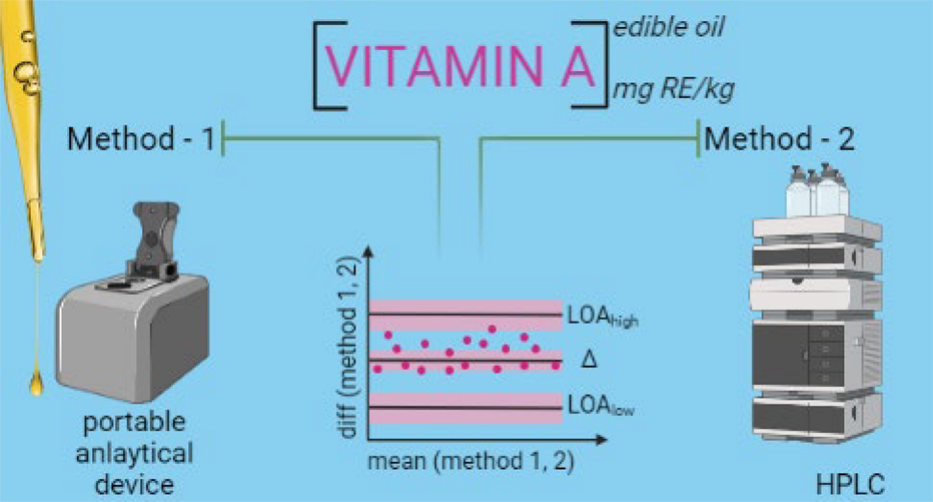

Vitamin A (VA) deficiency continues to be a major global health issue, despite measures to increase VA intake via consumption of staple foods such as edible oil. Portable quantitative and semiquantitative devices or test kits for internal quality control have the potential to overcome some of the limitations of traditional methods of testing, such as centralized laboratory, expensive equipment, and specially trained staff. This landscape analysis and comprehensive systematic mini-review catalogs and summarizes evidence on the analytical performance of portable quantitative and semiquantitative devices and test kits for the analysis of VA in edible oil. Studies or reports detailing the usability and validation of portable devices and/or test kits, as well as studies comparing device/test kit performance to a reference standard such as high-performance liquid chromatography (HPLC), were included. Identified devices and test kits were compared for performance versus the reference standard, usability, availability, and other characteristics. We identified four portable methods: two devices, the iCheck CHROMA and iCheck Chroma 3 from BioAnalyt; and two test kits, the QuickView from Bagco Enterprises and the Strategic Alliance for the Fortification of Vegetable Oils (SAFO) Test Kit by Badische Anilin and Soda Fabrik (BASF). Included studies reported the following: an internal validation of the portable method, a comparison of the portable method against a reference standard, a comparison of the portable method against another portable method, and several videos and company websites, which detailed device characteristics. iCheck CHROMA and QuickView quantified VA concentrations with high accuracy and precision compared to the reference standard for field-based quantification, were user-friendly, and provided results within 5 min. iCheck Chroma 3 requires more robust validation against a reference standard. We did not find data on internal validation or comparison against a reference standard for the current version of the SAFO test. Compared to QuickView and SAFO, the iCheck devices can transfer results to a hard drive or the Web, have an online order form for purchase, and meet a minimal set of criteria for point-of-need devices. iCheck, QuickView, and SAFO can quantify VA concentrations in the edible oils tested and determine whether a fortified oil meets country standards. Additional research is needed to validate these devices and test kits across additional oil types and document the ability to meet the minimal criteria for point-of-need devices suggested in this mini-review. Validation against a reference standard is required for SAFO. The limited number of portable methods available may be due to market saturation. Future market and use case analyses to inform the market size and utility of the different tests with publicly available data will allow new manufacturers, particularly those in lower-to-middle-income countries, to enter the market.

## Introduction

Vitamin A deficiency continues to be a major global health issue,^1^ despite measures to increase vitamin A intake via staple foods. Numerous quantitative techniques are available to determine vitamin A concentration in fortified staple products such as edible oils, sugar, and flour. However, most of these analytical techniques require access to a centralized laboratory for preparing samples and performing these tests using equipment such as high-performance liquid chromatography (HPLC) and gas chromatography (GC) coupled with ultraviolet–visible (UV–vis) detection. This equipment is expensive to procure and requires maintenance and consistent usage, reagents and preparation protocols that are resource-intensive, and skilled personnel to run.^1^ These methods are also time-consuming and potentially prohibitively expensive depending on the number of samples that need to be analyzed. In addition, trained laboratory personnel who can reliably operate such equipment are hard to find and retain in some settings. Alternative technologies have the potential to make gains in efficiency including costs, training, and maintenance compared to currently used equipment.

Portable quantitative devices or test kits (which output the exact concentration measured) and semiquantitative devices or test kits (which output results falling within a range of possible measurements, ascertained by matching to a color-coded legend) for internal quality control have the potential to overcome some of the limitations of traditional methods of testing. Some portable methods for determining the vitamin contents of fortified oils are available, and they may differ in their cost, accuracy, reliability, ease of use, and requirements of consumables/reagents required to perform the tests. In this mini-review we summarize features and performances of various portable quantitative and semiquantitative methods for vitamin A analysis and identify research and implementation gaps. This mini-review will enable current manufacturers to modify and improve their products and set design goals for new products that meet the current demands of industry, regulators, and other stakeholders.

In this mini-review, our objectives were to (1) catalog all portable semiquantitative and quantitative methods (devices and test kits) available for analysis of vitamin A in edible oil and (2) determine and compare the analytical performance of these devices for the analysis of vitamin A in edible oil. We also describe the roadmap for the development of such devices because they are much needed as global efforts for large scale food fortification gather steam.

## Results

**Figure 1:**
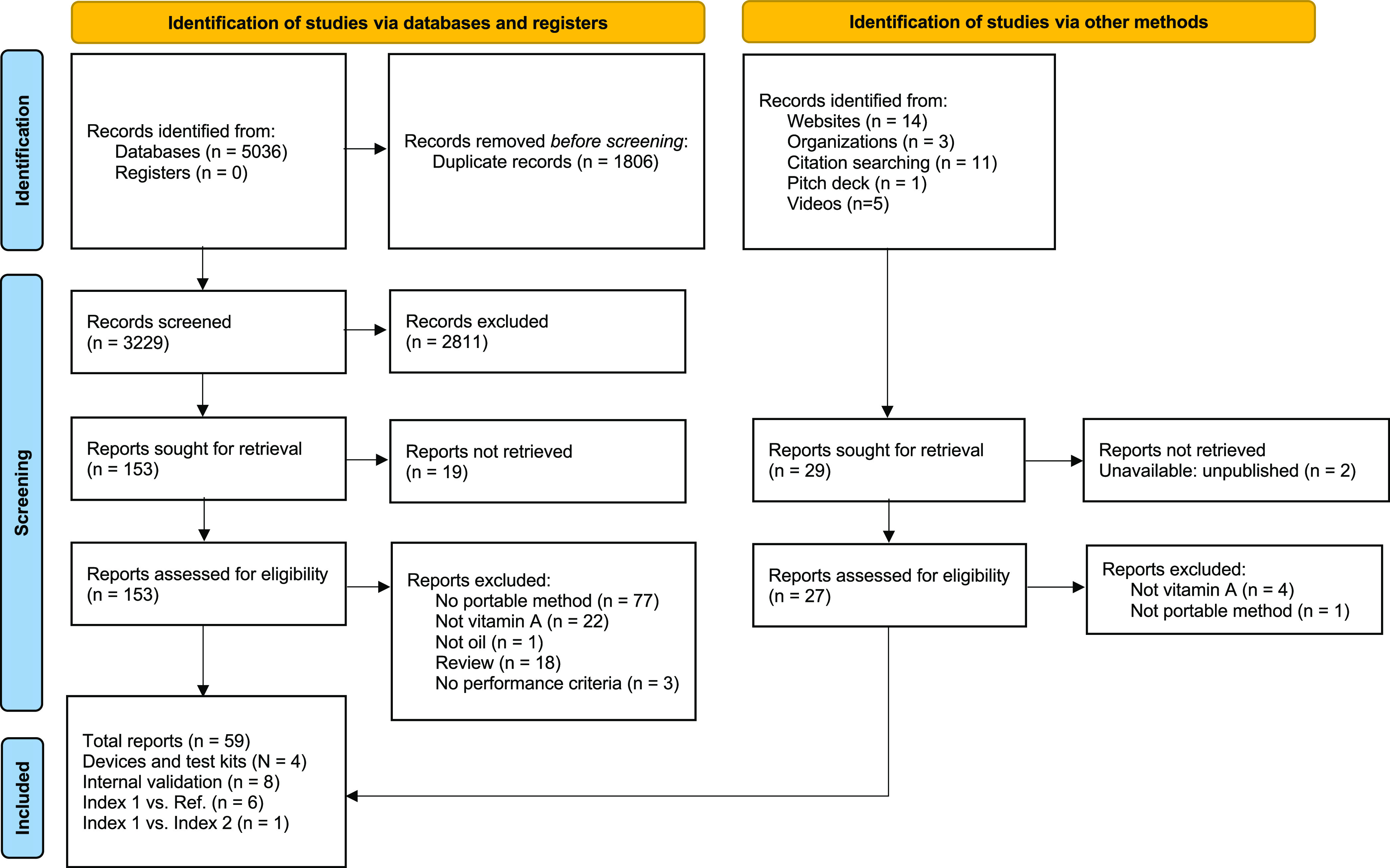
PRISMA^2^ diagram for study identification and screening. Adapted from the PRISMA Statement.

From 3258 records (after deduplication) identified from our searches via databases and other methods (Figure 1), we identified four portable methods: BioAnalyt’s iCheck CHROMA device and iCheck Chroma 3 device, the QuickView Vitamin A Test Kit by Bagco Enterprises, and the Strategic Alliance for the Fortification of Vegetable Oils (SAFO) Test Kit by Badische Anilin and Soda Fabrik (BASF) Test Kit for vitamin A in oil. The numbers and types of reports for each portable method are described below.

• iCheck CHROMA (referred hereafter as “iCheck CHROMA”) by BioAnalyt: Internal validation and comparison against a reference standard was described in two journal articles,^3^ one meeting abstract,^4^ one AOAC laboratory method,^5^ one conference proceedings,^6^ and a report from BioAnalyt.^7^ We also found one video describing step-by-step use of iCheck (https://youtu.be/v69kttryFOo). This version of iCheck is not currently advertised on the company website.

• iCheck Chroma 3 (referred hereafter as “iCheck Chroma 3”) by BioAnalyt: Internal validation and comparison against a reference standard was described in one meeting abstract^8^ and reports from BioAnalyt.^7^ One country survey of common cooking oils also utilized iCheck 3 and measured its precision.^9^ We also found two videos describing step-by-step use of iCheck 3 (https://youtu.be/zeCOdh8IqUs, https://www.youtube.com/watch?v=s2Kyg90qyz0). This updated version of iCheck CHROMA, iCheck Chroma 3, is currently advertised on the company website.^10^ iCheck Chroma 3 appears to be very similar to iCheck CHROMA but has improvements in operational range and estimation of some oils such as soy, as noted in a recent report.^7^

• QuickView (referred hereafter as “QuickView”) by Bagco Enterprises: Internal validation and comparison against a reference standard was described in one conference proceedings,^6^ one meeting abstract,^11^ and one report posted on the company website,^12^ (As a note, this conference proceedings^6^ contained comparisons of both iCheck and Quickview in separate reports within the proceedings; as such it is cited twice here.) We also identified one video describing step-by-step use of QuickView (http://bagcoent.com/gallery/videos/Quick%20View%20Instructional%20Video.mp4).

• SAFO Test Kit (referred hereafter as “SAFO”) by BASF: Internal validation was done on what appears to be a previous version of the device in one 2009 report^13^ and one 2007 method publication published by the organization.^14^ We also found one video describing step-by-step use of SAFO (https://youtu.be/-__5M4YF47o). The updated version of SAFO is currently advertised on the company website.^15^

All four portable methods assess vitamin A concentrations using the colorimetric Carr–Price reaction between vitamin A and antimony trichloride, which produces a blue color proportional to the vitamin A content.^16^ iCheck CHROMA and iCheck Chroma 3 are battery-operated portable photometers with optical design developed for the iEx ELAN reagent vial, containing reagents, and cuvette; the photometers are equipped with light emitting diode technology to measure vitamin A in oil. The QuickView test kit includes the equipment (test tubes, eye droppers) and reagents to combine and perform the reaction manually. Similarly, the SAFO test kit includes a set of pipets, vials, safety gear, and reagents to combine and perform the reaction manually.

Characteristics of the four portable methods, and reference standards such as HPLC, are described in Tables 1 and 2. Characteristics of each study are shown in Table 3. We show how well devices performed against manufacturer-advertised performance, using the ASSURED criteria as a guide, in Table 4. Finally, in Tables 5–8 we compared the specific performance criteria results from studies with internal validations as well as comparisons against a reference standard or other portable device.

**Table 1:** Device or Test Kit Characteristics: Affordability, Reagents Needed, Portability, Testable Oils, and Global Availability^a^

		reagents						
device/kit; no. tests per kit	costs^b^ (USD); pricing^c^	requirements	procurement; storage conditions and shelf life	device: portability; maintenance; other attributes	device/test kit: storage conditions and shelf life	acceptable oils	determines vitamin A levels around national fortification target level	manufacturer support available; global availability
iCheck CHROMA and iCheck Chroma 3;^d^ 100 tests per kit	device $6,730, test kit $800; $8.00 per test (on basis of test kit)	iEX ELAN disposable reaction vial, containing SbCl_3_ and CHCl_3_, 1 mL syringes, 0.8 mm × 16 mm needles	included in kit; 20–30 °C, no direct sunlight, upright; shelf life 12 months (10 million measurements possible)	portable (0.45 kg, 11 × 4 × 20 cm) with handle for carrying; NR; batteries are required	12 months at 25 °C; autocontrol to verify function of emitter and receptor	palm, soy, cottonseed,^e^ sunflower,^e^ corn,^e^ groundnut (peanut),^e^ rapeseed (colza), coconut, rice bran,^e^ vegetable (NR)	yes	website, email address, phone number and address; order directly from website; used in >80 countries and shippable globally
	++^f^	+++	+++	+++	+++	++	+++	+++
QuickView;^g^ 10 tests per kit	unclear if available for purchase	dropper, larger tubes (chloroform), smaller tubes (SbCl_3_), wire stirrer, instructions with color chart, tissue paper for breaking glass tubes safely	included in kit; NR	portable (dimensions and weight NR but appears small and lightweight); NR	NR	coconut, other light-colored cooking oils^e^	yes	unclear; contact information includes phone number, address, and email address, but email appears inactive since 2016; no indication on how to order
	+	++	++	+++	++	++	+++	+
SAFO,^h^ NR	pricing not published; estimated $0.05–$0.10 per test	water, dichloromethane, trichloroacetic acid, copper sulfate, ascorbic acid; sample of unfortified oil	included in kit; reagent (dichloromethane + trichloroacetic acid) cannot be exposed to >40 °C	portable (dimensions and weight NR; described as size of a laptop); NR; color-coded vials, jars, pipets	NR	sunflower,^i^ walnut,^i^ linseed,^i^ olive,^i^ safflower^i^	yes but must know target concentration in advance	website, contact form on website; yes
	+++	++	+++	++	+	++	++	++
HPLC coupled UV–vis^22^	$20,000–$50,000 per machine; $50–$100 per test	methanol, ethyl acetate, and isopropanol	purchase separately; depends on reagent	not portable; requires routine maintenance; NR	controlled conditions	palm, rapeseed, soy, coconut, and vegetable (unspecified)	yes	depends on manufacturer; yes
	+	++	++	+	+	+++	+++	+++
GC coupled UV–vis or MS	$10,000–$50,000 per machine; $25–$40 per test	methanol, ethyl acetate, and isopropanol	purchase separately; depends on reagent	not portable; requires routine maintenance; can be used for different analyses or where procurement of vials is difficult	controlled conditions	NR	yes	depends on manufacturer; yes
	+	++	++	++	+	+	+++	+++
UV spectro-photometer^22^	$1,000–$10,000; $7.50 per assay	dichloromethane, hexane	purchase separately; depends on reagent	not portable; requires routine maintenance	controlled conditions	not specified	yes	depends on manufacturer; yes
	++	+	++	++	+	++	+++	+++

a BASF, Badische Anilin and Soda Fabrik; GC, gas chromatography; GAIN, Global Alliance for Improved Nutrition; HKI, Hellen Keller International; IU, international units; MS, mass spectrometry; NR, not reported; ppm, parts per million; RE, retinol equivalents defined as 3.3 international units (IU) of vitamin A or as 1 μg; SAFO, Strategic Alliance for the Fortification of Oils and other Staple Foods; UNICEF, United Nations Children’s Fund; UV–vis, ultraviolet–visible detection; WFP, World Food Program.

b Costs are approximate.

c Published information cost per test, pricing not published, or proof-of-concept device/not commercially available.

d Information extracted from company website and included studies in refs (3,8, 10a, 10b, and 23).

e Not validated against a reference standard.

f +, not acceptable; ++, acceptable; +++, best.

g Information extracted from company website and included studies in refs (6, 12, and 21).

h Information extracted from company website and included studies in refs (13 and 20a).

i Tested, but not validated against a reference standard.

**Table 2:** Device or Test Kit Characteristics: Ease of Use/User Friendliness^a^

device/kit	training needs	instructions	calibration steps	sample preparation	steps	overall time required	results interpretation	recording results
iCheck CHROMA and iCheck Chroma 3^b^	1 h to <1 day training required	displays instructions at each step and company website includes user guide, instructional videos^c^^,^^d^^,^^e^ available on YouTube	precalibrated during manufacture; test using standard before using	warm, if solid, to maximum 50 °C to liquefy	four to five steps (blank measurement, sample injection, reaction and measurement, result display)	<5 min	exact concentration output on device	downloadable data including sample no., batch no., result (in mg of RE/kg or IU/g), date, time
	+++^f^	+++	+++	+++	+++	+++	+++	+++
QuickView^g^	<1 day training required: requires breaking glass to access reagents and requires	organization website includes instructional video^h^ and user guide	no calibration indicated in written instructions or video	no sample preparation required (no instructions for solid oil samples)	six steps (break big glass tube, add oil, break small glass tube, transfer materials, mix, reaction, comparison)	<5 min	within range of 5 mg of RE/kg	visually assess blue color change to match five reference colors (equivalent to different vitamin A concentrations from 5–25 mg of RE/kg)
	++	+++	+++	++	++	+++	++	++
SAFO^i^	<1 day training and lab experience required: requires extensive measuring and pipetting; color coding of equipment may limit use by individuals who are color-blind	company website includes instructional video; user manual not found	CuSO_4_ standards preparation required	no sample preparation required (no instructions for solid oil samples)	∼15 steps (see text); requires assumption of target vitamin A fortification level	<5 to 20 min, depending on training level	below, at, or above assumed target level	visually assess blue color change to match three reference colors, equivalent to being below, at, or above target fortification standard from light (below target), medium (target), or dark (higher than target)
	++	++	++	++	+	++	+	+
HPLC coupled UV–vis^22^	requires training from manufacturers, short courses, etc.	device manual available; various videos available	multiple-step flow rate calibration required	dilution with low-boiling solvent to fall in range of 1–30 mg/kg, homogenization, filtering	≥6 steps (dilution, homogenization, filtration, mobile phase, comparison to reference, washing)	≥65 min	exact concentration output on attached computer	chromatogram with quantified absorbance for vitamin A concentration
	+	+++	+	+	++	+	++	++
GC coupled UV–vis or MS	requires training from manufacturers, short courses, etc.	device manual available; various videos available	validate gas flow against predefined acceptance criteria, run dilutions, plot response times vs concentrations, construct calibration curves	dilution with low-boiling solvent, dissolve	four steps (sample dilution, dissolution, injection; ionization, filtration, detection)	≤60 min	exact concentration output on attached computer	chromatogram with quantified absorbance for vitamin A concentration
	+	+++	++	+	+++	+	++	++
UV spectrophotometer^22^	<1 day training and lab experience required	device manual available; various videos available	frequent calibration following manufacturer instructions to confirm calibration of monochromator	dilute sample in organic solvents; require blanks	eight steps (sample preparation, mixing, recording, correct absorbance rating, estimation)	∼20 min	exact concentration output on attached computer	absorption spectrum with quantified absorbance for vitamin A concentration
	++	+++	+	+	++	+++	++	++

a BASF, Badische Anilin and Soda Fabrik; GC, gas chromatography; GAIN, Global Alliance for Improved Nutrition; HKI, Hellen Keller International; IU, international units; MS, mass spectrometry; NR, not reported; ppm, parts per million; RE, retinol equivalents defined as 3.3 international units (IU) of vitamin A or as 1 μg; SAFO, Strategic Alliance for the Fortification of Oils and other Staple Foods; UNICEF, United Nations Children’s Fund; UV–vis, ultraviolet–visible detection; WFP, World Food Program.

b Information extracted from company website and included studies in refs (3, 8, 10a, 10b, and 23).

c iCheck Chroma: Self-training video: https://www.youtube.com/watch?v=v69kttryFOo.

d iCheck Chroma 3: How to control your device, 1/2: https://www.youtube.com/watch?v=zeCOdh8IqUs.

e iCheck Chroma 3: How to measure your sample, 2/2. https://www.youtube.com/watch?v=s2Kyg90qyz0.

f +, not acceptable; ++, acceptable; +++, best.

g Information extracted from company website and included studies in refs (6, 12, and 21).

h QuickView instructional video: http://bagcoent.com/gallery/videos/Quick%20View%20Instructional%20Video.mp4.

i Information extracted from company website and included studies in refs (13 and 20a).

**Table 3:** Description of Included Studies Validating the Portable Method and/or Comparing against a Reference Standard^a^

author year; report type	device	manufacturer	oil sample tested	source of oil sample	test location (field/lab, country)	fortification standard described/noted	reference method	ref
Bullecer 2016;^b^ conference proceedings	iCheck CHROMA	Bioanalyt GmbH	coconut	commercial	NR (Philippines)	Philippine Food Fortification Law: 12–23 mg of RE/kg	HPLC	(4)
Makhumula 2016; conference proceedings	iCheck Chroma 3	Bioanalyt GmbH	vegetable oil (unspecified)	commercial	NR (Uganda)	Uganda: 25 mg of RE/kg	UV-S	(8)
Maramag 2016 and Castro 2016;^b^ website, conference proceedings	QuickView	Bagco Enterprises	coconut	commercial	NR (Philippines)	Philippine Food Fortification Law: 12–23 mg of RE/kg	HPLC	(11, 12)
NCP 2016;^b^ conference proceedings	iCheck CHROMA	Bioanalyt GmbH	coconut	commercial	NR (Philippines)	Philippine Food Fortification Law: 12–23 mg of RE/kg	HPLC	(6)
	QuickView	Bagco Enterprises	coconut	commercial	NR (Philippines)	Philippine Food Fortification Law: 12–23 mg of RE/kg	HPLC	(6)
Renaud 2013; journal	iCheck CHROMA	Bioanalyt GmbH	rapeseed, soy, groundnut	commercial	NR (France)	N/A	HPLC, UV/vis	(3b, 7, 23)
Rohner 2011; journal	iCheck CHROMA	Bioanalyt GmbH	palm	households/commercial	field (Côte d’Ivoire); laboratory (Germany)	Côte d’Ivoire: 6.4–9.6 mg of RE/kg, i.e., 8 mg of RE retinyl palmitate/kg of oil	HPLC, UV/vis	(3a, 5, 7)
GAIN 2018; coutnry survey	iCheck Chroma 3	Bioanalyt GmbH	sunflower, cotton, palm olein, palm, soybean, vegetable, rapeseed, corn, blended, peanut, red palm	commercial	Burkina Faso	11–24 mg of RE/kg	none	(9)
BioAnalyt 2020; company report (internal data)	iCheck Chroma 3	Bioanalyt GmbH	soy	NR	NR	N/A	none	(7)

a HPLC, high performance liquid chromatography; NCP, Nutrition Center of the Philippines; NR, not reported; RE, retinol equivalents defined as 3.3 international units (IU) of vitamin A or as 1 μg; UV-S, ultraviolet spectrophotometry.

b NCP reported the same QuickView and iCheck validation studies that were also reported by Bullecer et al.,^4^ Maramag et al.,^12^ and Castro et al.^11^ Maramag et al.^12^ and Castro et al.^11^ are the same report: the former is posted on the company website and the latter was accepted at the Micronutrient Forum 2016 conference.

**Table 4:**
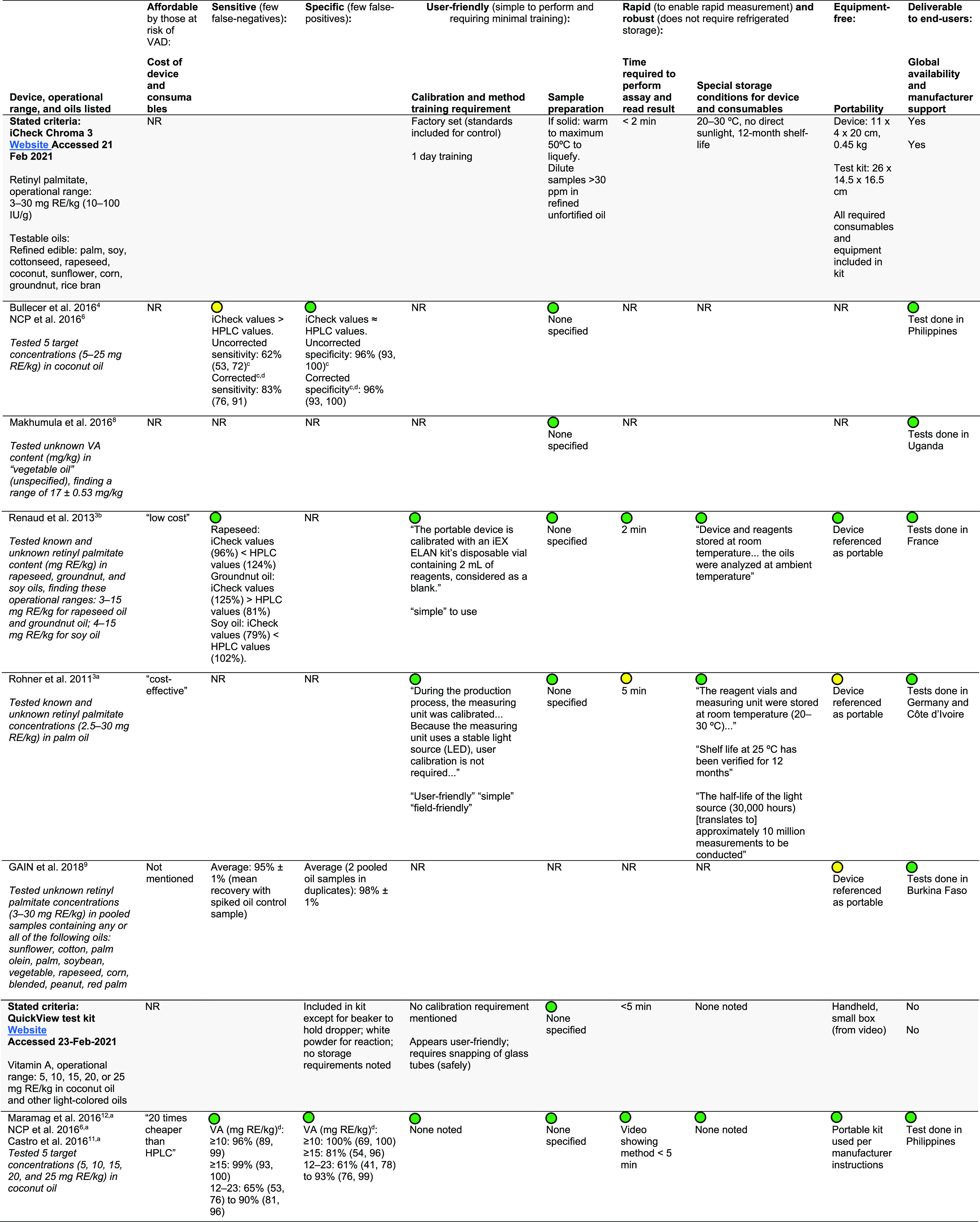
Assessment of Devices and Test Kits^a^ against Manufacturer-Reported Performance, by ASSURED^b^ Criteria^e^

a Only studies that mention any of the ASSURED criteria are included.

b ASSURED criteria have been adapted from ref (24).

c Ranges are given as 95% confidence intervals.

d “Corrected” sensitivity: iCheck values were corrected by subtracting the mean difference between iCheck and HPLC from the actual reading.

e HPLC, high-performance liquid chromatography; n/a, not available; NR, not reported; RE, retinol equivalents. Green = matches stated criteria; yellow = somewhat matches stated criteria; red = completely different between manufacturer’s stated criteria and study reporting.

**Table 5:** iCheck CHROMA: Device Performance for Palm, Rapeseed, and Groundnut Oils^a^

	palm oil^3a,5,7^			rapeseed oil^3b,7,23^		groundnut oil^3b,7,23^	
	validation	index 1^b^ vs reference^c^^,^^d^	index 1^b^ vs index 2^e^	validation	index 1 vs reference^d^	validation	index 1 vs reference^d^
validation of portable device							
no. samples	200	189	200	15	15	15	15
*R*^2^, coefficient	0.996, Spearman	0.92, Spearman	0.94, Spearman	NR	0.981^g^	NR	0.988^g^
regression equation	*y* = 0.99*x* ± 0.12	*y*_HPLC_ = 0.83*x* + 0.08	*y*_field_ = 0.93*x* + 0.08	*y* = 1.24*x* ± 0.84	*y*_HPLC_ = 0.96*x* + 0.48	*y* = 1.25*x* ± 0.36	*y*_HPLC_ = 0.81*x* + 1.09
vitamin A concn levels tested (mg of RE/kg); replicates	2.8, 5.6, 6.5,^h^ 11.2, 13.0,^h^, 16.8, 22.4, 25.0,^h^ 28.0; 3			3.0, 7.5, 15.0, 22.5, 30.0; 3	3.0, 7.5, 15.0, 30.0; 5	3.0, 7.5, 15.0, 22.5, 30.0; 3	3.0, 7.5, 15.0, 30.0; 5
operational range (mg of RE/kg) (LOD_low_ to LOD_high_)	2.5–30	0–16	0–16	3–15		3–15	
accuracy	N/A	0.1% difference in adequately fortified samples		N/A	RMSE: index 1 = 3.99; reference = 0.61	N/A	RMSE: index 1 = 5.49; reference = 2.25
precision							
intra-assay % CV	3.2–7.1%; 1.7–10.6%^h^			8.6%; 2.1%^f^		7.0; 1.8%^f^	
interassay % CV	1.4–8.8%; 0.2–3.3%^h^						
interobserver % CV	3.8–4.8%; 1.1–3.9%^h^						
Bland–Altman mean bias LOA or mean difference for portable device (mg of RE/kg)		LOA_low_ = −1.24; LOA_high_ = 2.53	LOA_low_ = 0.60; LOA_high_ = 1.99		mean difference: ∼3		mean difference: ∼4.5

a LOA, limits of agreement, calculated as Δ – 2*s* = LOA_low_ and Δ + 2*s* = LOA_high_, where Δ is the mean of the difference between the two methods and *s* is the standard deviation of this difference. LOD, limit of detection, where LOD_low_ is the lowest concentration of detection and LOD_high_is the maximum concentration of detection. NR, not reported; RE, retinol equivalents defined as 3.3 international units (IU) of vitamin A or as 1 μg; RMSE, root mean squared error.

b Portable test in Germany (laboratory setting).

c Reference test in Germany (laboratory setting).

d Reference test is HPLC.

e Portable test in Côte d’Ivoire (field setting).

f At vitamin A concentrations of 3 and 15 mg of RE/kg, respectively.

g Coefficient of determination.

h These concentrations and accompanying results were measured by AOAC;^5^ nonsuperscripted concentrations were only included in Rohner et al.^3a^

The iCheck devices, QuickView, and SAFO were described as generally user-friendly, requiring less than 1 day’s training; ideal for field settings without a fully equipped laboratory; able to provide useful data indicating whether an oil has been fortified to a target level or range; and portable and easy to set up in a new location (Tables 1 and 2). Affordability of any of the devices was not readily ascertained from public information. All four devices quantified vitamin A concentration in oil that would allow ascertainment of that oil meeting fortification standards. iCheck CHROMA and iCheck Chroma 3 have been reported to be used across the world by the manufacturer, and this is supported by studies in Cameroon,^17^ Ghana,^18^ and Indonesia^19^ reporting having used iCheck CHROMA and iCheck Chroma 3. No surveys or reports were found showing use of QuickView in any country. We found reports describing the distribution and use of SAFO in Tanzania, Bolivia, and Indonesia.^20^

### Advantages and Disadvantages

The main advantage of these methods are their portability and lack of sample preparation, in comparison to the reference standard, HPLC coupled with UV–vis detection. All four devices—iCheck CHROMA, iCheck Chroma 3, QuickView, and SAFO—can yield results in under 5 min, from adding the sample to the test kit to reading the result, depending on training level and experience. A disadvantage of iCheck is that waste materials (such as hazardous, corrosive reagents such as antimony trichloride) require implementing an appropriate waste-management system.^3^ Though not mentioned in reports, this disadvantage would apply to QuickView and SAFO as well, which also require hazardous materials. Following are more specific advantages or disadvantages that were heterogeneous among the portable methods, by characteristic.

#### Ability to Test a Range of Oil Types

Although the iCheck manufacturer (BioAnalyt) website describes the working range as 3–30 mg of RE/kg of any of the oils listed, it should be noted that this range depends on the type of oil; for example, as shown by Renaud et al. and Rohner et al., the working range for rapeseed, groundnut, and soy oils are 3 or 4 to 15 mg of RE/kg,^3b^ while the range for palm oil was as stated 3–30 mg of RE/kg.^3a^ This is a limitation of iCheck, as the manufacturer lists several acceptable oil types for which validations against a reference standard have not been done (including cottonseed, sunflower, corn, groundnut, and rice bran). According to a 2020 report,^7^ iCheck CHROMA also underestimates vitamin A content in soy oil; this limitation has been overcome in the updated version of the device, iCheck Chroma 3, which shows linearity between known retinyl palmitate concentrations in oil and those which were measured by iCheck Chroma 3, leading to an operational range of 0–30 mg of RE/kg (*R*^2^ = 0.97).

QuickView has been validated against HPLC in coconut oil but no other light-colored oils, as advertised, and SAFO has not been validated against HPLC in any oils.

#### Ease of Use

Of all the portable methods, iCheck, being a contained device, required the fewest number of steps, four to five, to perform a test, including blank measurement, sample injection, reaction and measurement, and result display. Subsequently, there would be less potential for error in measurement or other techniques compared to the QuickView and SAFO test kits, which involve 6 and 15 steps, respectively.

If users had some laboratory experience, iCheck training took 1 h in one study,^3a^ and instructional videos show the simplicity and user-friendliness of using the device, such as minimal handling of reagents.

From the instructional video and written instructions, QuickView appeared to require minimal training as well, though the amount of training was not quantified in any report. One safety concern is controlled glass breaking (using tissue paper as protection), which was necessary to access the chemical solutions; this could be dangerous without proper instruction.^21^ QuickView required six steps to complete one test, including breaking of reagent tube 1 glass, adding the oil, breaking of reagent tube 2 glass, mixing, reaction, and reading the result.

A 2019 video (https://youtu.be/-__5M4YF47o) shows step-by-step actions for performing a test using SAFO, which involves double or triple the number of steps for setting up, mixing the reagents, and adding the oil sample compared to iCheck devices and QuickView. One disadvantage of the SAFO kit is that the vials, pipets, and jars included are all color-coded, using red, light green, dark green, light blue, dark blue, and yellow for various steps—this will exclude individuals with certain forms of color-blindness from performing tests and increase the possibility of user error in keeping track of which color is used at which step. Having the jars simply labeled with text (e.g., “step 1: dichloromethane + trichloroacetic acid”) would mitigate this issue. Another disadvantage is that it appears one would need samples of both unfortified *and* fortified oils, as it is necessary to add certain amounts of both oils to the jar to measure the amount of vitamin A in the fortified oil. The biggest disadvantage of the SAFO kit is the required assumption of the user regarding the level of vitamin A fortification in the sample, which informs how much of each oil (fortified and unfortified) is added to the reaction. This would be based on from where the oil was sampled, on a country-by-country basis. In other words, performing tests of completely unknown vitamin A concentrations in oils does not appear to be possible. Finally, similar to the inconsistency of the written instructions versus graphical instructions in the 2009 report, one of the steps in the 2019 video was performed clearly using a dark *blue* pipet, while the concurrent audio instructions reference using a dark *green* pipet (at 4:33–4:54). Overall, the many steps involved in BASF/SAFO analyses may result in greater user error and mismeasurements, and this test may be only applicable to measuring vitamin A content in oils with a known range or target level of fortification, not unknown oils.

#### Calibration Requirement

Studies indicated that the iCheck devices are precalibrated during manufacture so that no calibration or vitamin A standard is required, saving time; however, the user should first use a blank in the device before adding an oil sample. It appeared that QuickView also required no calibration. In contrast, SAFO requires preparation of copper sulfate standard solutions to aid in comparisons.

#### Results Interpretation

An advantage of both iCheck devices is their ability to output exact results, minimizing error in accurately estimating vitamin A concentrations. In contrast, the QuickView and SAFO test kits require subjective interpretation of results by matching the blue color in the test tube after the reaction is complete to a reference guide showing different shades of blue. Specifically, the QuickView manual includes a reference for five shades of blue to compare with the blue color in the tube (gradually getting darker from 5 to 25 mg of RE/kg in increments of 5 mg of RE/kg). SAFO includes a reference with three shades of blue (light, medium, dark) representing whether vitamin A concentrations are below (light), at (medium), or above (dark) the target concentration assumed when performing the test. As mentioned above, BASF/SAFO therefore cannot ascertain unknown vitamin A content from oil samples.

#### Recording Data

A major advantage of iCheck CHROMA and iCheck Chroma 3 is the ability to download the quantified data via a universal serial bus (USB) into a laptop as a text file, in two different units for vitamin A concentration. In contrast, QuickView and SAFO necessitate the user to record results separately, with the instructional video showing the technician writing the results down using pen and paper, leading to repetitive data entry upon digitizing later and concomitant risk of recording or transcription error.

#### Ordering and Availability

Finally, iCheck Chroma 3 is available and simple to order online via the manufacturer. We did not identify a website to order QuickView, the manufacturer of which, Bagco Enterprises, appears to have assigned rights and interests to Casnar Corp. in 2016. Attempts to contact this company were unsuccessful. The SAFO kit does not have an ordering webpage but does have a contact form.

### Included Validation and Comparison Studies

Studies were conducted in Côte d’Ivoire, France, Germany, the Philippines, Burkina Faso, or Uganda, or the country was not reported (Table 3). iCheck CHROMA was validated with and shown to able to test a range of oils, but we did not identify validation data for every oil advertised by the manufacturer. Only coconut oil was used in validation of and testing using QuickView. The results of iCheck CHROMA and iCheck Chroma 3 were published in two peer-reviewed journal articles,^3^ one laboratory method paper,^5^ one report from the manufacturer,^7^ one country survey by an organization,^9^ and four conference papers (two of which reported the same study).^4,6,8,23^

The internal validation of QuickView and comparison to a reference standard were reported in one conference proceedings, one meeting abstract, and one report posted on the company website (all reporting the same data and results).^6,11,12^

Table 4 shows a comparison of how the devices performed in individual studies against stated performance criteria from manufacturer websites, using the ASSURED (*A*ffordable, *S*ensitive, *S*pecific, *U*ser friendly, *R*apid, *E*quipment-free, *D*eliverable to end users) criteria.^24^ Most criteria as reported by studies were consistent with stated criteria from manufacturers. Much of the criteria could not be ascertained from conference abstracts or other shorter reports. We did not include the SAFO device in Table 4 because we did not find any studies or reports using the current version of the test kit.

Tables 5–Table 8 show performances of the iCheck devices or QuickView test kits across included studies for measuring our target outcome, mass of vitamin A per mass of oil (mg of RE/kg). We assigned the portable device as the “index test”, and if there were two portable devices compared to each other, we designated these “index test 1” and “index test 2”. The reference standard for the majority of studies was HPLC.

Rohner and colleagues^3a,5,7^ validated iCheck CHROMA in the laboratory (“index 1”) and compared it both to a reference standard and to a field-based test (“index 2”) in Côte d’Ivoire (Table 5). All other studies compared the iCheck CHROMA or Chroma 3 to the reference only. Operational ranges for vitamin A concentration by iCheck CHROMA varied by oil, between a lower limit of 2.5–5 mg of RE/kg and an upper limit of 15–30 mg of RE/kg, as shown by Renaud and colleagues^3b,7,23^ (Table 5, Table 6). In testing known vitamin A concentrations in palm, rapeseed, groundnut, and soy oils, iCheck CHROMA performed well with *R*^2^ values ranging from 0.92 to 0.988 against the reference standard. Bland–Altman plots to evaluate agreement between the two measurement techniques^25^ indicated good agreement with small mean differences for palm, rapeseed, groundnut, and soy oils. For coconut oil, iCheck CHROMA readings were higher than the reference for the majority of samples (Table 6).

**Table 6:** iCheck CHROMA: Device Performance for Soy and Coconut Oils^a^

	soy oil^3b,7,23^		coconut oil^4,6,11^	
	validation	index 1 vs reference^b^	validation	index 1 vs reference^b^
validation of portable device				
no. samples	15	15	100^d^	NR^d^
*R*^2^, coefficient	NR	0.983^c^	NR	NR
regression equation	*y* = 0.79*x* ± 1.18	*y*_HPLC_ = 1.02*x* ± 0.22	NR	NR
vitamin A concn levels tested (mg of RE/kg); replicates	3.0, 7.5, 15.0, 22.5, 30.0; 3	3.0, 7.5, 15.0, 30.0; 5	5, 10, 15, 20, 25; NR	NR; NR
operational range (mg of RE/kg) (LOD_low_ to LOD_high_)	4–15		5–27.8	
accuracy	N/A	RMSE: index 1 = 4.91; reference = 0.40	N/A	
precision				
intra-assay % CV	NR;^e^ 2.4%^f^			
interassay % CV			3.5–7.3%	
interobserver % CV			1.3–14.7%	
Bland–Altman mean bias LOA or mean difference for portable device (mg of RE/kg)		mean difference: ∼ –4		mean difference: 1.9 ± 2.3 (95% CI: −2.6, 6.5)

a LOA, limits of agreement, calculated as Δ – 2*s* = LOA_low_ and Δ + 2*s* = LOA_high_, where Δ is the mean of the difference between the two methods and *s* is the standard deviation of this difference. LOD, limit of detection, where LOD_low_ is the lowest concentration of detection and LOD_high_ is the maximum concentration of detection. NR, not reported; RE, retinol equivalents defined as 3.3 international units (IU) of vitamin A or as 1 μg; RMSE, root mean squared error.

b Reference test is HPLC.

c Coefficient of determination.

d While a sample size of 100 was given, it is unclear if this was the total sample size (50 samples per iCheck, 50 samples per HPLC) or 100 samples per each method.

e Not reported because LOD for soy oil is >3 mg of RE/kg.

f At vitamin A concentrations of 3 and 15 mg of RE/kg, respectively.

iCheck Chroma 3 remains to be compared to reference standards with larger numbers of samples and replicates (Table 7). In testing a vegetable oil (not specified),^8^ iCheck Chroma 3 reported smaller average concentration milligrams of RE per kilogram compared to UV-S, but it is unclear if either result was closer to the actual vitamin A value, which was not reported; authors cited needing to validate UV-S against HPLC to ascertain this information. From internal data^7^ via the manufacturer, testing soy oil with iCheck Chroma 3 resulted in a good performance against known vitamin A concentrations, but it was not tested against HPLC. Finally, in a country survey,^9^ iCheck Chroma 3 was able to detect oils with adequate amounts of vitamin A in fortified oils, but oils were various and pooled together for analysis, making individual oil data not possible to ascertain.

**Table 7:** iCheck Chroma 3: Device Performance^a^

	vegetable (unspecified) oil^8^			
	validation	index 1 vs reference^b^	soy oil^7^ validation	cooking oil,^c^ validation
validation of portable device				
no. samples	10	10	NR	214 (pooled to 91 composite samples)
*R*^2^, coefficient	NR	NR	0.97, NR	NR
regression equation	NR	NR	*y* = 0.99*x* + 1.14	NR; duplicates times 4
vitamin A concn levels tested (mg of RE/kg); replicates	average concn: 17 ± 0.5; 10	average concn:^d^ 24 ± 3.2; 10	3–30	
operational range (mg of RE/kg) (LOD_low_ to LOD_high_)	NR	NR	0–30	NR
accuracy	N/A		N/A	N/A
precision				
intra-assay % CV	3%^e^		NR	0.9–2.7%
interassay % CV			NR	NR
interobserver % CV			NR	NR
Bland–Altman mean bias LOA or mean difference for portable device (mg of RE/kg)		not done		

a LOA, limits of agreement, calculated as Δ – 2*s* = LOA_low_ and Δ + 2*s* = LOA_high_, where Δ is the mean of the difference between the two methods and *s* is the standard deviation of this difference. LOD, limit of detection, where LOD_low_ is the lowest concentration of detection and LOD_high_ is the maximum concentration of detection. NR, not reported; RE, retinol equivalents defined as 3.3 international units (IU) of vitamin A or as 1 μg.

b Reference test is HPLC.

c Including some or all of the following:^9^ sunflower, cotton, palm olein, palm, soybean, vegetable, rapeseed, corn, blended, peanut, red palm.

d Reference test is UV-S.

e Coefficient of variation: unclear if intra-assay, interassay, or interobserver.

The QuickView validation study^4,6,11,12^ was conducted with coconut oil and found that higher concentrations of oil vitamin A yielded values closer to HPLC-tested oil vitamin A concentrations (Table 8). Authors concluded that QuickView was most suitable for identifying a minimum oil vitamin A concentration threshold of 10–15 mg of RE/kg, as measured by high sensitivity and specificity; this was suitable for the current Philippines fortification standard of 12–23 mg of RE/kg.

**Table 8:** QuickView: Device Performance^a^

	coconut oil^4,6,11,12^	
	validation	index 1 vs reference^b^
validation of portable device		
no. samples	100	NR^c^
*R*^2^, coefficient	NR	0.66, NR
regression equation	NR	*y*_HPLC_ = 5.04*x* + 0.93
vitamin A concn levels tested (mg of RE/kg); replicates	5, 10, 15, 20, 25; NR	5, 10, 15, 20, 25; NR
operational range (mg of RE/kg) (LOD_low_ to LOD_high_)	NR	NR
accuracy	N/A	
precision		
intra-assay % CV		
interassay % CV		
interobserver % CV		
Bland–Altman mean bias LOA or mean difference for portable device (mg of RE/kg)		mean difference: −4.036

a Notes: LOA, limits of agreement, calculated as Δ – 2*s* = LOA_low_; Δ + 2*s* = LOA_high_ where Δ*i*s the mean of the difference between the two methods and s is the standard deviation of this difference. LOD, limit of detection, where LOD_low_ is the lowest concentration of detection and LOD_high_ is the maximum concentration of detection; NR, not reported; RE, retinol equivalents defined as 3.3 international units (IU) of vitamin A or as 1 μg.

b Reference test is HPLC.

c While a sample size of 100 was given, it is unclear if this was the total sample size (50 samples per QuickView, 50 samples per HPLC) or 100 samples per each method.

Information on measurement error and reliability and repeatability of results (intra-assay % CV, interassay % CV, and interobserver % CV) where reported are also shown in Tables 5–8. Reported values were fairly low, indicating minimal variability for these metrics.

## Research and Development Plan and Minimal Set of Features of a Point-of-Need Analytical Device for Vitamin A Analysis in Edible Oil

In summary, there are currently four portable methods—iCheck CHROMA device, iCheck Chroma 3 device, the QuickView test kit, and the SAFO test kit—to test for vitamin A content in edible oils, in addition to the reference standards such as UV/vis spectroscopy alone or coupled with HPLC or GC separation. The limited number of portable methods available may be due to market saturation.

### Gaps and Recommendations

We found a lack of published or available information regarding both internal validation and comparisons of the portable methods against a reference, particularly for iCheck Chroma 3 and SAFO. BioAnalyt suggests its iCheck devices are appropriate for a range of edible oils, but we only found studies testing some of these oils. With the findings by Renaud et al. showing that different oils had different operational ranges, this is a major gap in the data, even if iCheck Chroma 3 appears to have addressed this issue (information only from manufacturer's internal data, not from a third-party report). Similarly, QuickView was tested only in coconut oil (no other light-colored oils). There were no recent studies using the current SAFO device showing its performance in any oils.

In addition, studies describing details of the user experience of any device were few, limiting our ability to compare this domain across studies.

The reports^3,4,6,8,11,12,23^ comparing the portable methods described in this review to a reference were all proof-of-concept or pilot studies. The majority of the reports were published as conference abstracts or proceedings, limiting our ability to ascertain methodological quality using, for example, the Quality Assessment of Diagnostic Accuracy Studies (QUADAS) framework.^26^ Only one study clearly tested a portable device in a field setting, which is the target location for using these devices.^3a^

On the basis of the available data regarding portable method attributes and performance, summarized above, we concluded that iCheck CHROMA, iCheck Chroma 3, QuickView, and SAFO acceptably quantify vitamin A content in edible oil to determine whether the oil meets the fortification standards. All four portable methods are appropriate for field use and are user-friendly; choosing which method to use depends on the user’s goal. The iCheck devices are the only devices able to meet all three of the following goals and have the largest range of testable oils, but some still require testing. If the goal is accurate quantification of unknown vitamin A amounts in oil, iCheck CHROMA or iCheck Chroma 3 should be used. iCheck 3 should be used in the case of measuring soy oil. If the goal is to analyze oil with an unknown vitamin A content within a range, QuickView (if QuickView is available for purchase) is appropriate. If the goal is to assess whether an oil with an assumed target level of vitamin A fortification is meeting the standard, and unfortified oil samples are available for use, then SAFO is appropriate.

Future research on portable methods for vitamin A assessment in fortified oil should be performed using appropriate diagnostic test accuracy (DTA) methodology to ensure applicability of study results and minimize risk of bias (QUADAS).^26^ In addition to validating portable devices against a reference standard, it will be useful to compare two or more portable devices with each other to guide researchers and other entities engaged in this work. All fortified edible oils should be tested to determine interoil differences in the portable method performance. Studies should include a field-based comparison, in addition to lab-based device performance, for real-world applicability. Finally, future study designs should include considering the Index Test and Reference Standard related domains of the QUADAS 2 framework, as well as potentially adapting or replacing the domains related to human participants with a new domain on oil sample selection methodology and flow. These changes will be helpful to ascertaining risks of bias in the studies and the applicability of the portable device.

### Minimal Set of Criteria for Point-of-Need Devices

**Figure 2:**
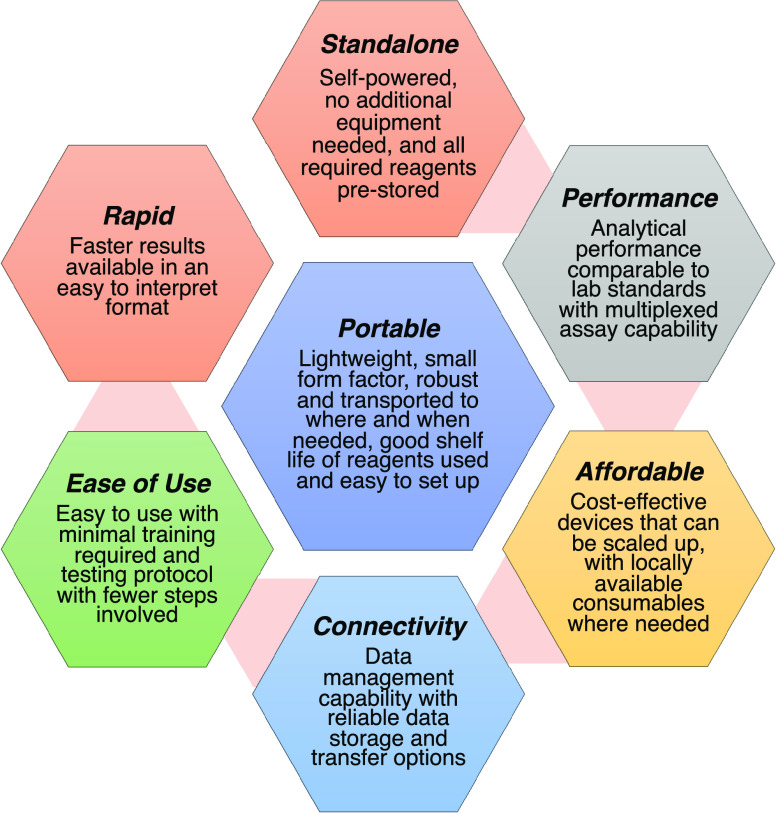
Minimal set of criteria for point-of-need devices.

On the basis of the studies included in this mini-review, we have developed a minimal set of seven criteria for point-of-need devices (Figure 2). iCheck Chroma 3 appears to meet all criteria. QuickView meets all but number 6 and partially meets number 7.

The device should be

(1) lightweight with a small form factor for easy transport to where and when it is needed
(2) standalone without needing additional equipment, self-powered, prestore all the required reagents for the test, and use common reagents that are available on the market
(3) easy to use with minimal processing steps in the protocol and require minimal training effort
(4) comparable in analytical performance to the current lab standards with capability to test various biological samples
(5) affordable and scaled up with locally available consumables where needed
(6) able to connect to the internet or an external hard drive with a built-in data management system to allow the test results to be reliably stored and transferred
(7) able to output test results quickly and present in a format that is easy to interpret

## Design and Development of a Portable Spectrophotometer for Quantification of Vitamin A in Oil

Numerous spectrophotometer prototype designs for low-cost, educational purposes using pocket digital cameras, photodiode detectors/LEGO blocks, and 3D printable smartphone spectrophotometers have been reported.^27^ In this section, we briefly describe the design and development including validation of a portable spectrophotometer device coupled with a mobile app that can be applied for the quantification of vitamin A in oil samples and also meets the ASSURED criteria. Quantification of vitamin A is based on the blue color reaction as described by Carr and Price.^16^ The underlying reaction is based on double bonds of the retinol molecule reacting with antimony trichloride (SbCl_3_) in chloroform resulting in the formation of retinylic and anhydroretinylic cations,^28^ appearing intense blue^16^ as described by Carr and Price. The absorption of the blue color is proportional to the concentration of the solution when measured at 610 nm with the portable spectrophotometer device. Main components of the portable spectrophotometer include a spectrophotometer chip to measure absorption, an LED as a stable light source, a commercially available microcontroller such as Raspberry Pi Pico, or Arduino Nano or a low-cost, credit card size computer such as Raspberry Pi 4 model B, and a 9 V battery to power the device. A 3D printed enclosure is used to assemble the various components along with a cuvette holder to accommodate a standard cuvette.

**Figure 3:**
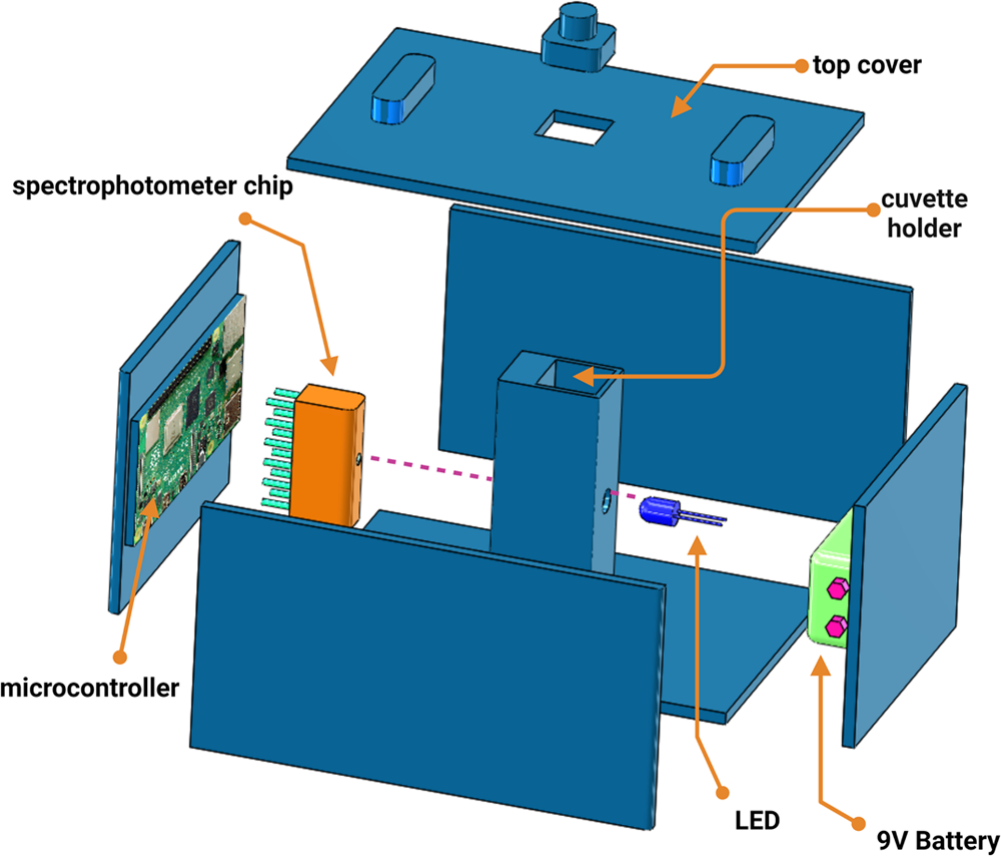
Exploded view of the portable spectrophotometer with a 3D printed casing for housing the various components. Created with BioRender.com.

**Figure 4:**
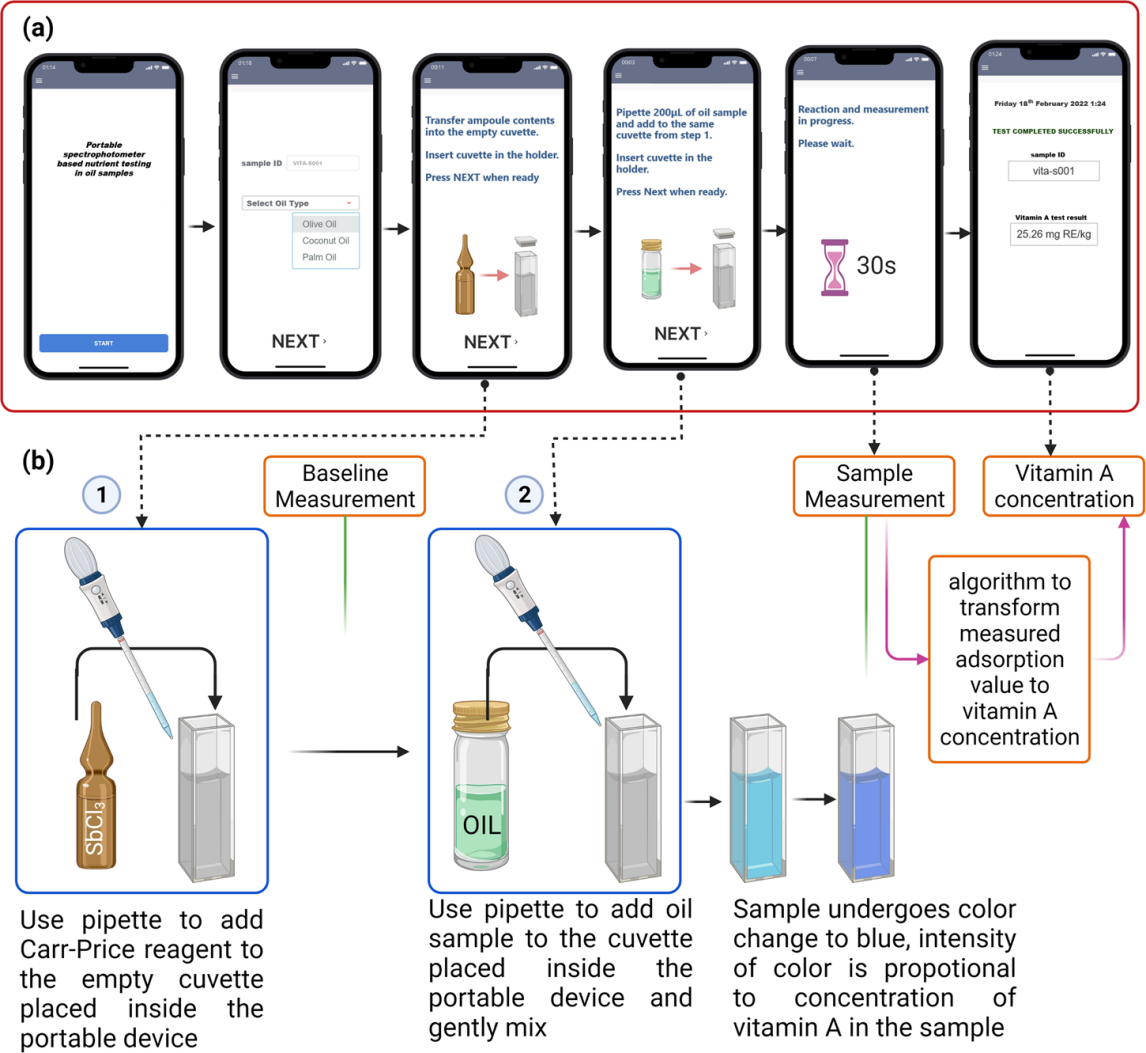
(a) Screenshots of the mobile app. (b) Sample processing steps involved in quantification of vitamin A in oil samples on the portable spectrophotometer device. Created with BioRender.com.

Figure 3 shows an exploded view of the portable spectrophotometer with the various components assembled within the 3D printed enclosure. A mobile app can be designed to communicate wirelessly with the spectrophotometer via Bluetooth or Wi-Fi and also provide step-by-step instructions to the user performing the test. Test kit reagent and components include the following: an ampule with fixed volume Carr–Price reagent (saturated solution of antimony trichloride (SbCl_3_) in chloroform (CHCl_3_)) required per test, empty cuvettes, and a pipet. Figure 4 shows screen designs of the mobile app along with the various steps involved in the testing protocol. Briefly, the testing procedure involves use of the mobile app to collect sample information and selection of the type of oil sample. The mobile app provides instructions to guide the user to add the reagent to the cuvette and place it in the cuvette holder to make a baseline measurement. This is followed by the addition of a specific volume of oil sample to the same cuvette and mixing of the sample by gentle forward and reverse pipetting. After the sample addition step is completed by the user, the mobile app wirelessly controls the portable spectrophotometer to make a measurement after 30 s. Based on the absorbance measurement, the vitamin A concentration of the test sample is predicted by the mobile app from the prestored calibration curve for the sample type. Test results can be displayed on the mobile app screen and also on a standalone computer interface with the option of transferring the acquired data via text message/email or to a cloud database.

### Performance Characterization of Portable Spectrophotometer

**Figure 5:**
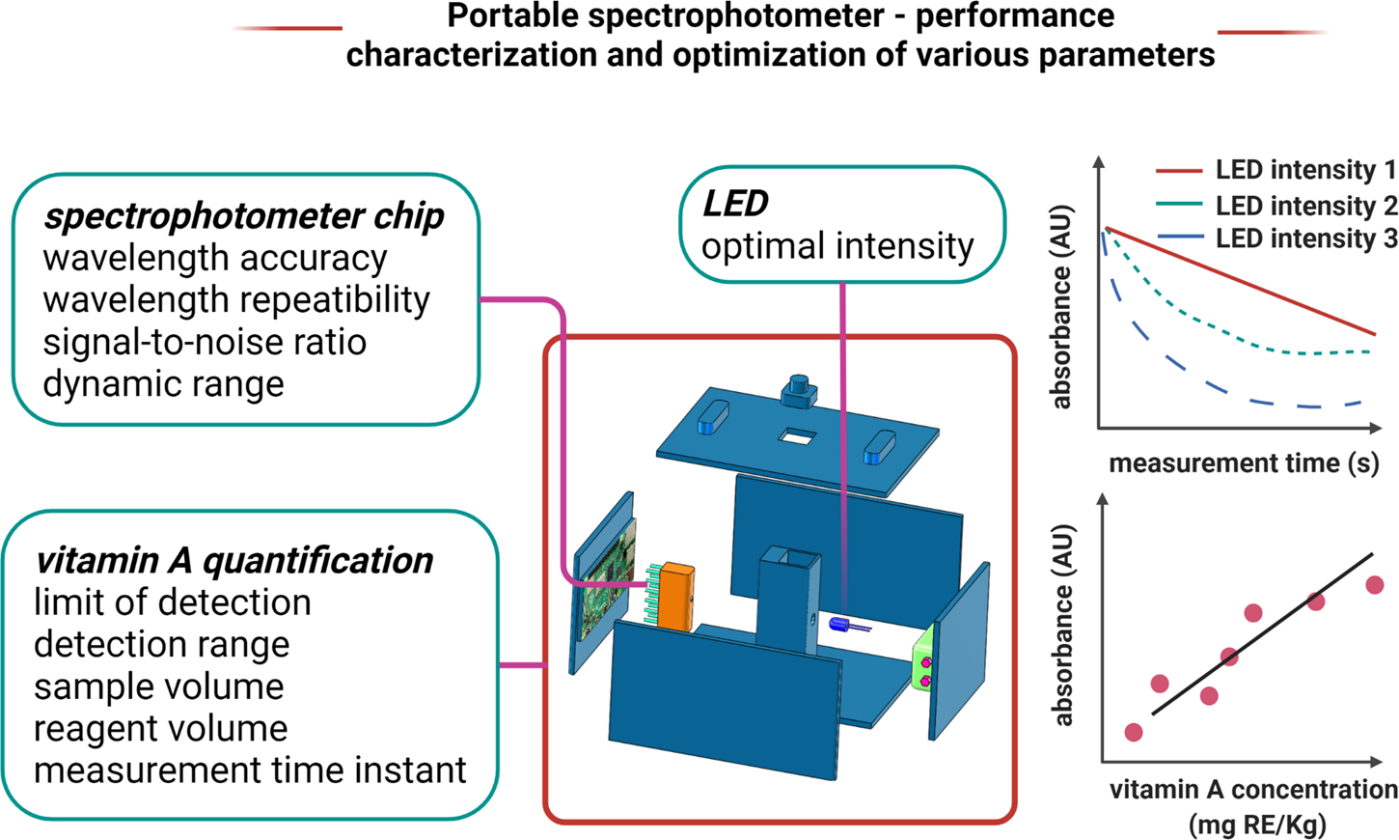
Schematic summarizing the various performance parameters to be optimized and performance characterization plots for portable spectrophotometer. Created with BioRender.com.

The spectrometer chip is a critical component of the system, and performance parameters such as wavelength accuracy, wavelength repeatability, and signal-to-noise ratio are important to estimate the quality of measurements that can be achieved with the device. These performance parameters can be experimentally determined if the chip does not have any internal calibration features with detailed calibration specifications provided by the manufacturer. In order to characterize the portable spectrophotometers for quantification of vitamin A, preliminary testing with reference standard oil samples of known vitamin A concentrations can be tested to determine the calibration curve, detection range, and optimal sample and reagent volumes per test. It has been previously reported^29^ that the intensity of the blue color developed upon mixing vitamin A and SbCl_3_ reagent is unstable and is affected by intensity of the illumination source (LED light) applied during the measurement proces. The optimal intensity of the LED light source and the optimal time instant at which measurement is to be made can be determined by investigating the kinetics of the Carr–Price reaction with the portable spectrophotometer. Figure 5 summarizes the various performance parameters to be optimized for various components of the portable spectrophotometer and the types of performance characterization plots.

### Validation of Portable Spectrophotometer against HPLC Reference Standard

**Figure 6:**
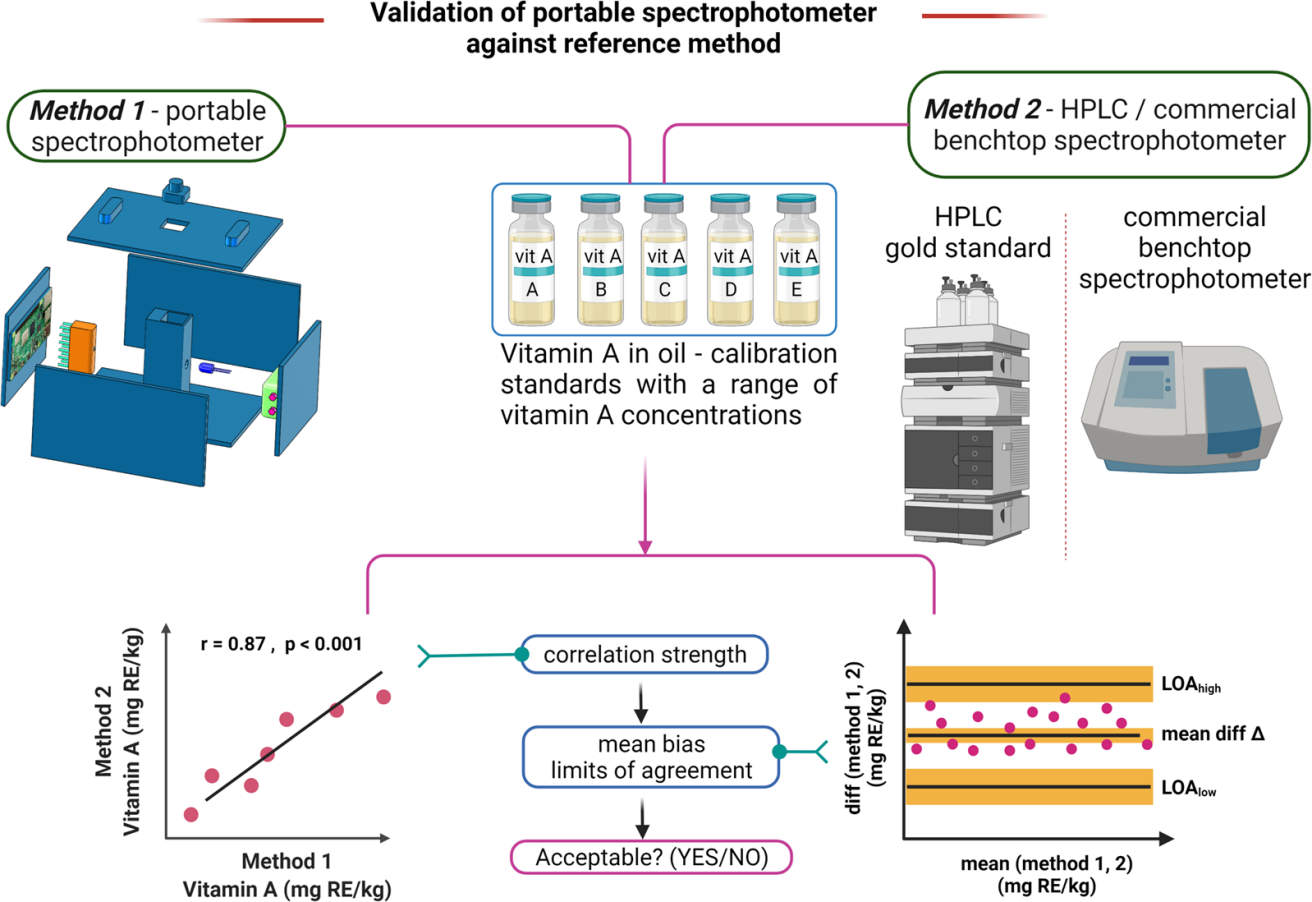
Validation of portable spectrophotometer against a reference method and statistical analysis to determine acceptability for a target vitamin A quantification application. Created with BioRender.com.

The performance of the portable spectrophotometer for the quantification of vitamin A in various oil samples can further be characterized by comparing absorption results for a range of concentrations with commercial benchtop spectrophotometers or comparing vitamin A test results against the high performance liquid chromatography (HPLC) reference method.^30^ Validation tests can be performed by testing with test samples of known vitamin A concentrations spanning the desired detection range and comparing results between the portable spectrophotometer (method 1) and HPLC or a commercial benchtop spectrophotometer (method 2) (Figure 6). To compare the results from the two methods, statistical methods such as regression analysis and Bland–Altman analysis can provide data on correlation and agreement between the two methods. The limits of agreement data from Bland–Altman analysis can be effective in deciding whether the portable spectrophotometer would be a good fit for an application depending on the maximum acceptable limits of agreement and correlation strength observed with a reference method. The portable spectrophotometer described here meets all of the ASSURED criteria, and we hope the design and validation approach discussed here could be useful in guiding the development of various analytical devices for application in quantification of vitamin A in oil samples.

## Conclusions

In this mini-review, we identified four portable methods for measuring vitamin A in oil. iCheck CHROMA and iCheck Chroma 3 provided acceptable approximations of vitamin A concentrations in rapeseed, groundnut, soy, palm, and coconut oils in comparison to HPLC. The QuickView test kit allowed vitamin A concentration assessment most applicable for reaching a minimum threshold for coconut oil. The SAFO test kit can determine vitamin A content as long as the user can assume the target level of vitamin A concentration in the oil, and this kit requires study of its performance in comparison to a reference standard. Further research in developing and validating portable devices should consider adding comparisons of different oils and adding biomarkers such as β-carotene for example in the case of red palm oil and consider portable devices, the minimal set of criteria for point-of-need devices outlined in this mini-review, and methodological quality assessment.

## Methods

In December 2020, we conducted a standardized search of the literature indexed in five databases (MEDLINE, EMBASE, World Health Organization Global Index Medicus, Scopus, Web of Science, and Agricola) with no restrictions on language, location, or date of publication. We designed a search strategy for MEDLINE (PubMed) and translated the search strategy for the remaining databases with guidance from the evidence synthesis specialists at Mann Library, Cornell University. Table S1 shows our MEDLINE search strategy that was adapted for other databases (Supporting Information). We also used an internet search to identify other sources such as manufacturer websites and patents and consulted with industry experts for more information.

Portable quantitative or semiquantitative devices and test kits that measure vitamin A in edible oil were eligible for this review. Inclusion criteria for studies assessing these devices included any study describing the portable device’s performance characteristics and/or internally validating the performance of the device or test kit in measuring vitamin A concentration [measured as vitamin A palmitate; 1 μg of retinol activity equivalent (RAE) equals 1 retinol equivalent (RE, which is defined as 3.3 international units of vitamin A or as 1 μg of retinol), 1 μg of retinol, 2 μg of β-carotene in oil, 12 μg of β-carotene in mixed foods, or 24 (12–26) μg of other provitamin A carotenoids in mixed foods^31^]. We also included studies that compared the performance of a portable method with a reference standard such as HPLC or GC coupled with UV–vis detection or mass spectrometry. Studies involving human participants (e.g., observational studies or randomized-controlled trials) were considered if there was an embedded method comparison of interest.

Devices or test kits were excluded if they were not portable, did not measure vitamin A, or did not measure vitamin A in edible oil. Studies were also excluded if they did not describe performance characteristics or compare the device to the reference standard.

We also identified company websites and reports describing the device. Research articles which reported using the portable method for a study or survey or organizational reports that mentioned use of the portable method in the field but did not mention any performance criteria such as precision or accuracy results were not included as studies but are cited as appropriate in this mini-review. For example, a reference mentioning the per-assay cost of the portable method but not mentioning precision results was cited for the assay cost information but was not added to our list of included studies.

We contacted any manufacturers who had a product on the market and authors who have published in this area to request raw data or more information as needed.

We assessed the following device/test kit characteristics:

- ease-of-use/user friendliness: the device can be used by those who are not highly trained in food or chemical analysis, including sample preparation or overall time required for analysis from adding the sample to reading the result
- target setting: the device can be used in an environment which is not restricted to a fully equipped testing laboratory
- target range: the device provides data to indicate whether an oil has been fortified at a target level or within a target range
- availability and affordability of device and consumables: the device is affordable in terms of capital investment relative to “fixed-equipment” laboratory test methods such as UV–visible spectrophotometry and HPLC high-performance liquid chromatography
- portability: the device or test kit is portable/mobile and easily able to be set up in a new location
- special storage conditions
- shelf life of device and consumables
- availability of manufacturer’s support and capacity to supply a global demand: either directly or through a distribution network; ability to purchase the test online easily through an order form
- ability to test a range of oil types, including darker oils or unrefined oils

We also summarized validation studies reporting the portable method’s internal precision as well as accuracy and precision compared against the reference standard, HPLC, GC, or UV–vis. Performance criteria included the following:

- internal validation: intra-assay, interassay, and interobserver variation, correlation or *R*^2^ value, accuracy, and linearity
- comparison to reference: intra-assay, interassay, and interobserver variation, correlation or *R*^2^ value, accuracy, linearity, Bland–Altman median bias limits of agreement, observed agreement, mean difference, sensitivity, specificity

## Supporting Information Available

Example of our PubMed search strategy (PDF)
