# Supplementary material for: Portable Devices for Measurement of Vitamin A Concentrations in Edible Oil: Field Readiness of Available Options
Source: ACS Omega. 2022 May 17;7(21):17502–18. doi: 10.1021/acsomega.1c07181 (PMC9161250; doi:10.1021/acsomega.1c07181)
Supplement: Supplementary file 1 — ao1c07181_si_001.pdf [file ao1c07181_si_001.pdf]

Supporting Information

Portable Devices for Measurement of Vitamin A Concentrations in Edible Oil – Field Readiness of Available Options

Samantha L. Huey[1], Jesse T. Krisher[1], David Morgan[2], Penjani Mkambula[2], Balaji Srinivasan[1], Bryan M. Gannon[1], Mduduzi N.N. Mbuya[3], Saurabh Mehta[1,4]*

[1] Division of Nutritional Sciences, Cornell University, Ithaca, NY, United States

[2] Department of Large Scale Food Fortification, The Global Alliance for Improved Nutrition, Geneva, Switzerland

[3] The Global Alliance for Improved Nutrition, Washington, DC, United States

[4] Institute for Nutritional Sciences, Global Health, and Technology (INSiGHT), Cornell University, Ithaca, NY, United States

***Corresponding Author:**

Saurabh Mehta, MBBS, ScD

Division of Nutritional Sciences

Cornell University

Martha Van Rennselaer Hall, Suite 3101A

Ithaca, NY, 14853, United States

Phone: +1 (607) 255-2640

Email: smehta@cornell.edu

Keywords: vitamin A; retinol; portable devices; fortification; edible oils; device; field devices; test kit

Table S1. MEDLINE (PubMed) search strategy

#	Search string	Records: December 16, 2020
**1**	vitamin a[mesh]	44,757
**2**	vitamin a[tiab] OR retinol[tiab] OR retinyl[tiab]	35,049
**3**	#1 OR #2	60,517
**4**	Palm oil[mesh] OR soybean oil[mesh] OR cottonseed oil[mesh] OR peanut oil[mesh] OR rapeseed oil[mesh] OR coconut oil[mesh] OR rice bran oil[mesh] OR plant oils[mesh]	37,121
**5**	Palm oil[tiab] OR soybean oil[tiab] OR cottonseed oil[tiab] OR peanut oil[tiab] OR rapeseed oil[tiab] OR coconut oil[tiab] OR rice bran oil[tiab] OR plant oil*[tiab] OR “edible oil*”[tiab] OR safflower oil[tiab] OR canola oil[tiab] OR sunflower oil[tiab] OR palm kernel oil[tiab] OR red-palm oil[tiab] OR olive oil[tiab] OR corn oil[tiab] OR fish oil[tiab] OR margarine[tiab]	41,490
**6**	#4 OR #5	64,982
**7**	#3 AND #6	762
